# IgM Immunoglobulin Influences Recovery after Cervical Spinal Cord Injury by Modulating the IgG Autoantibody Response

**DOI:** 10.1523/ENEURO.0491-19.2021

**Published:** 2021-09-08

**Authors:** Antigona Ulndreaj, Pia M. Vidal, Nicole Forgione, James Hong, Michael G. Fehlings

**Affiliations:** 1 Division of Genetics and Development, Krembil Brain Institute, University Health Network, Toronto, Ontario M5T 2S8, Canada; 2 Institute of Medical Science, Faculty of Medicine, University of Toronto, Toronto, Ontario M5T 2S8, Canada; 3 Department of Surgery, Faculty of Medicine, University of Toronto, Toronto, Ontario M5T 2S8, Canada; 4 University of Toronto Spine Program, University of Toronto, Toronto, Ontario M5T 2S8, Canada; 5 Department of Basic Science, Biomedical Science Research lab, Faculty of Medicine, Universidad Católica de la Santísima Concepción, Concepción, Chile 4090541

**Keywords:** functional recovery, IgG-autoantibody response, IgM immunoglobulin, spinal cord injury

## Abstract

Spinal cord injury (SCI) results in the development of detrimental autoantibodies against the lesioned spinal cord. IgM immunoglobulin maintains homeostasis against IgG-autoantibody responses, but its effect on SCI recovery remains unknown. In the present study we investigated the role of IgM immunoglobulin in influencing recovery after SCI. To this end, we induced cervical SCI at the C6/C7 level in mice that lacked secreted IgM immunoglobulin [IgM-knock-out (KO)] and their wild-type (WT) littermate controls. Overall, the absence of secretory IgM resulted in worse outcomes as compared with WT mice with SCI. At two weeks after injury, IgM-KO mice had significantly more IgG antibodies, which fixed the complement system, in the injured spinal cord parenchyma. In addition to these findings, IgM-KO mice had more parenchymal T-lymphocytes as well as CD11b+ microglia/macrophages, which co-localized with myelin. At 10 weeks after injury, IgM-KO mice showed significant impairment in neurobehavioral recovery, such as deteriorated coordination, reduced hindlimb swing speed and print area. These neurobehavioral detriments were coupled with increased lesional tissue and myelin loss. Taken together, this study provides the first evidence for the importance of IgM immunoglobulin in modulating recovery after SCI and suggests that modulating IgM could be a novel therapeutic approach to enhance recovery after SCI.

## Significance Statement

The present study provides novel evidence for the protective role of IgM immunoglobulin in SCI. Using a clinically relevant mouse model of SCI at the cervical level (C6/C7), we show that deficiency of IgM immunoglobulin results in impaired neurobehavioral recovery, coupled with increased lesion size, less white matter sparing, and enhanced deposition of complement-fixing IgG antibodies in the spinal cord. These data provide evidence for the necessary role of IgM immunoglobulin in spontaneous recovery during cervical SCI and warrant more research into the therapeutic effect of IgM administration after SCI.

## Introduction

There is compelling preclinical and clinical evidence that spinal cord injury (SCI) results in the development of autoantibodies against spinal cord tissue as well as the induction of systemic autoantibodies (Mizrachi et al., 1983; Petrova et al., 1993; Hayes et al., 2002; Ankeny et al., 2006, 2009; Davies et al., 2007; Zajarías-Fainsod et al., 2012; Ulndreaj et al., 2017; Hergenroeder et al., 2018). Although the role of autoantibodies in human SCI remains largely unknown, research in animal models indicates that autoantibodies have a detrimental effect post-SCI (Ankeny et al., 2006, 2009). Thus, understanding the mechanisms that lead to the development of autoantibodies after SCI and limiting their detrimental effects could improve recovery in this devastating condition.

We recently found that rats with cervical SCI had an expanded IgM response, as shown by early extravasation of IgM immunoglobulin in the lesioned spinal cord and by chronically increased counts of IgM plasma cells in the spleen after injury (Ulndreaj et al., 2017). Research in other diseases suggests that IgM immunoglobulin is a key regulator of IgG-mediated autoimmunity (for review, see Ehrenstein and Notley, 2010). As such, some IgM autoantibodies have been shown to have protective effects in autoimmune conditions such as atherosclerosis and systemic lupus erythematosus (SLE). For example, increased levels of IgM autoantibodies against oxidized low-density lipoprotein (ox-LDL) and phosphorylcholine were associated with protection against atherosclerosis when adjusted for age, gender, smoking, total cholesterol levels and other factors (Su et al., 2006). Also, a lower ratio (below 0.8) of serum IgG/IgM autoantibodies against ds-DNA was correlated with an absence of nephritis in patients with SLE (Förger et al., 2004). IgM immunoglobulin is also protective against infections (Boes et al., 1998a; Baumgarth et al., 2000, 2005), which are critically important in patients with SCI, as they can modulate neurologic recovery (Failli et al., 2012; Kopp et al., 2017) and constitute the most frequent co-morbidity (DeVivo et al., 1999). Lastly, IgM is the carrier and a negative regulator of apoptosis inhibitor of macrophage (AIM) protein (Hiramoto et al., 2018; Miyazaki et al., 2018). AIM, also known as CD5L/Spalpha/API6, is associated with autophagy and efferocytosis (Sanjurjo et al., 2015), and it is one of the most highly upregulated genes early on after SCI (Zhu et al., 2017). Importantly, disruption of AIM’s signaling by deletion of its receptor CD36 has been shown to result in improved neurobehavioral recovery following SCI in mice (Zhu et al., 2017).

Given this background, we posited that the expanded IgM response observed previously in our cervical SCI study could be a compensatory mechanism to limit the production of detrimental IgG autoantibodies observed post-SCI (Ulndreaj et al., 2017). Therefore, we hypothesized that mice deficient in IgM immunoglobulin will have impaired recovery after SCI. By inducing cervical SCI in a mouse model that lacks the secretory form of IgM immunoglobulin [IgM-knock-out (KO); Boes et al., 1998b], we show that lack of IgM results in the enhanced deposition of IgG antibodies in the spinal cord early on (two weeks) after injury coupled with a widespread inflammatory response in the spinal cord. Chronically (10 weeks after injury), these IgM-KO mice presented with worse locomotor recovery, increased lesion size and less white matter preservation. Thus, this study provides evidence for the protective role of IgM immunoglobulin following cervical SCI.

## Materials and Methods

### Mice

All animal procedures were approved by the University Health Network Animal Care Committee and experiments were conducted according to the Guide to the Care and Use of Experimental Animals designed by the Canadian Council of Animal Care. Adult female B6;129S4-*Ighm^tm1Che^*/J mice were obtained from The Jackson Laboratory (JAX stock #003751; Boes et al., 1998b) and were subsequently bred at the Krembil Discovery Research Animal Facility. In these mice, the μ_s_ exon encoding the secreted form of the μ heavy chain, and its three downstream poly(A) sites, are replaced by the μ_m_ exon which encodes the constant region of the mu4 exon and the exon encoding the membrane-bound form of IgM (Boes et al., 1998b). For this study we bred heterozygotes and used mice that were homozygous for the μ_m_ exon (here referred to as IgM-KO or KO) and littermate mice that were homozygous for the μ_s_ exon [here referred to as IgM-wild-type (WT) or WT]. As a result, the IgM-KO mice are deficient of secretory IgM immunoglobulin, although they express surface bound IgM and can class-switch into IgG immunoglobulin (Boes et al., 1998b).

### C6/C7 SCI model

SCI at the C6/C7 level was induced in female adult mice as previously established in our laboratory (Forgione et al., 2017). Blood was collected via the saphenous vein from all animals 24 h before surgery to establish baseline (BSL) levels of serum immunoglobulin. To induce C6/C7 SCI, anesthesia was achieved by isoflurane (2%) delivered in a 1:1 mixture of O_2_/N_2_O throughout the procedure. Following skin incision and muscle retraction between C5 and T2, the C6 and C7 laminae were removed. A micro-rongeur was carefully used to widen the laminectomized area and a 5.25-g modified aneurysm clip was applied to compress the C6 level for 40 s. Next, a small piece of Surgifoam (Ethicon) was used to cover the injured area, followed by muscle and skin suturing. Mice received subcutaneous buprenorphine (0.05 mg/kg, twice daily for 3 d) and saline (1 ml daily for 7 d) to prevent pain and dehydration, respectively. Sham mice underwent all above procedures, except for clip compression. All groups were housed in standard mouse cages (a maximum of five animals per cage), which were placed in a warm chamber of 27°C and 50% humidity. In mice with SCI, bladders were expressed manually three times a day until bladder function returned to normal. Lastly, antibiotics (Clavamox) were delivered in drinking water for two weeks to prevent infection postoperatively. Animals were sacrificed at 2 and 10 weeks after injury. The two-week study was replicated whereby two independent experimenters (A.U. and N.F.) performed surgeries, performed behavioral tests and collected tissues for subsequent analyses. Since the study cohorts showed similar neurobehavioral outcomes (data not shown) for all subsequent assessments in the study, samples were retrieved randomly from the two cohorts to minimize bias.

### Behavioral analyses

Mice underwent locomotor and grip strength tests to assess their neurologic recovery at selected time points after SCI. At weeks 1–3 post-SCI, we used the Basso Mouse Scale (BMS) test and the grip strength test to assess locomotion and forelimb grip recovery, respectively (Forgione et al., 2017). For BMS testing, animals were placed individually in an open field and allowed to locomote for 5 min during which time they were scored using a previously-established 10-point scale (0–9; Basso et al., 2006). For the grip strength test, the SDI Grip Strength System model DFM-10 (San Diego Instruments) was used. In this test, forelimb motor function was assessed based on the animal’s ability to pull a metal grid located on an electronic grip strength meter. Each trial consisted of five separate pulls. The highest and lowest forces were omitted and the remaining three were averaged, as previously reported (Forgione et al., 2017).

At weeks 4–10 post-SCI, we used the CatWalk XT 10.6 system (Noldus Information Technology) to assess neurologic recovery in the forelimbs and hindlimbs. Of note, the CatWalk test could not be performed before the fourth week after injury as the animals were unable to complete the test consistently. In this study, we collected a minimum of three appropriate runs per animal at 4, 8, and 10 weeks post-SCI. However, for analysis, we only considered runs demonstrating a complete gait pattern, where maximum speed variation within each run was <50%, and the average speed between runs was not significantly different (Batka et al., 2014; Vidal et al., 2017). Additionally, data from animals that were unable to perform the test reliably for reasons that were not SCI-related (such as grooming, exploring the device during the test, etc.) were not collected for analysis. The parameters selected for analysis were stride length, print area, swing speed, swing time, step sequence regularity and average body speed, as previously reported (Forgione et al., 2017). All above tests were performed by one experimenter who was blinded to the groups.

### Tissue collection and processing

At 2 and 10 weeks after injury, animals received an overdose of inhalant isoflurane and tissues were collected in the following order. Blood was collected from the heart and was left to clot at room temperature (RT) for at least 30 min for serum preparation for ELISA to confirm the absence of secretory IgM in KO mice and for the quantification of serum IgG and IgM. Next, animals were transcardially perfused with ice-cold PBS followed by isolation and processing of tissues of interest.

The bladder and a piece of the colon from each animal were weighed, homogenized in sterile PBS, serially diluted and then cultured in standard LB agar plates for 24 h at 37°C. The lungs were fixed in 4% paraformaldehyde (PFA) for 30 min and then in formalin overnight, followed by embedding, sectioning and staining for histology assessment as previously described (Ulndreaj et al., 2017). Spinal cords were fixed in 4% PFA-PBS for 3 h, then incubated in 10% sucrose (w/v)-PFA for 3 h, followed by an overnight incubation in 20% sucrose (w/v)-PBS at 4°C. A 1-cm-long section of the cervical cord centered at the injury epicenter was further selected for sectioning and 30-μm-thick cryosections were stored at −80°C before use for immunofluorescence staining.

### Quantitative lesion analysis

Lesion analysis was performed at 2 and 10 weeks post-SCI. Thirty-micrometer-thick sections at 600-μm intervals were stained with luxol fast blue (LFB) and hematoxylin-eosin (HE). Stereology was conducted using a Nikon Eclipse E800 microscope with a motorized stage and StereoInvestigator software (MBF Biosciences). Lesion volume and spared gray/white matter volume were quantified for each section using the Cavalieri Estimator function. Spared gray matter was considered as the non LFB-stained area displaying normal cytoarchitecture and easily distinguishable neuronal cell bodies. Spared white matter was considered as the LFB-stained area with normal architecture and non-lesional tissue. As previously reported (Forgione et al., 2017), here we defined lesional tissue as areas with loss of normal cytoarchitecture, absence of neuronal cell bodies, presence of fibrotic and/or glial scar tissue, infiltrated immune cells, as well as presence of vacuoles and loss of LFB stain (in gray matter).

### Immunofluorescence assessments in the lesioned spinal cord

#### IgG deposition in the injured spinal cord

IgG deposition in the lesioned spinal cord was assessed by quantifying the % area of IgG+ immunofluorescent signal at 2 and 10 weeks post-SCI, following a previously reported protocol (Ulndreaj et al., 2017). Of note, this method is not sensitive to the intensity of the detected signal, but rather it semi-quantifies the spread of the IgG content on the tissue. Thus, IgG deposition refers to the degree of IgG spread on the tissue, expressed as percent of the total spinal cord section.

In brief, following air-drying and rehydration in PBS, cryosections were blocked with 5% goat serum + 1% BSA + 0.3% Triton X-100 in PBS for 1 h at RT. Cy3-conjugated goat anti-mouse F(ab’)_2_ IgG antibody (1:250, Jackson ImmunoResearch) and 4′,6-diamidino-2-phenylindole (DAPI; 1:250) were added to the slides and incubated overnight at 4°C. Of note, a F(ab’)_2_ IgG detecting antibody was used to minimize labeling of Fc receptors in the injured parenchyma. Sections were washed four times with PBS and slides were cover-slipped with Mowiol mounting medium (Sigma). To avoid staining variability, all samples from the same time point were stained simultaneously.

Samples were selected randomly for imaging and images were acquired as close together as possible. For the two-week time point, a Nikon Eclipse E800 microscope at 10× magnification was used. The threshold was set such that the brightest sections (injury epicenter) were not overexposed yet signal from the least bright sections could still be detected. A similar procedure, although using a Nikon eclipse Ti C2+ inverted confocal microscope, was followed for the 10-week time point. At both time points, all settings were kept constant during imaging. Signal from spinal cord sections, excluding the dura, was measured with the appropriate plugin in ImageJ software and assessed under identical settings. The IgG+ area was expressed as % of the total area of the spinal cord section.

#### Complement-fixing IgGs

Complement fixation by deposited IgGs in the spinal cord was assessed in spinal cord sections with the highest IgG signal. These sections, which were located −1200, −600, and +600 μm from the injury epicenter, were co-stained for IgG and complement C3b protein and the amount of complement fixing IgGs was semi-quantified based on the IgG+/C3b+ positive immunofluorescent area. Briefly, sections were first stained for IgG as described above. Next, they were incubated with anti-mouse C3b-FITC antibody (1:100, Cederlane, clone 11H9 recognizing C3/C3b/iC3b) and DAPI (1:250) diluted in 5% goat serum + 1% BSA + 0.3% Triton X-100 in PBS, overnight at 4°C. Lastly, sections were washed, cover-slipped and imaged with a Nikon eclipse Ti C2+ inverted confocal microscope, as described above. For each section, the area of positive immunofluorescence from IgG alone, C3b alone and IgG and C3b signal was estimated with ImageJ and expressed as % of the total area of the spinal cord section. Then, the obtained values for the three sections (representing distances −1200, −600, and +600 μm from the injury epicenter) were averaged per animal.

#### Serum IgG and IgM immunoglobulin levels

Serum IgG and IgM immunoglobulin was quantified by ELISA developed in-house. Briefly, standard 96-well plates were coated with 2 μg/ml of either capturing anti-mouse IgG antibody (Abcam) or anti-mouse IgM antibody (Abcam, ab 9175) O/N at 4°C. Following one wash with PBS, plates were blocked with 4% goat serum in PBS for 2 h at RT. Next, serum diluted in 4% goat serum was added at 1:50,000 (for detection of IgG) and at 1:10,000 (for detection of IgM) and incubated for 4 h at RT. Mouse IgG (Sigma, 15381) or IgM (BioLegend, 401601, clone MM-30) purified from mouse serum was serially diluted in 1:2, starting from 10 μg/ml, to create a standard curve in every plate ranging from 10 μg/ml to 9.8 ng/ml. Plates were then washed with 0.05% Tween in PBS and incubated for 30 min at RT with anti-mouse IgG-HRP (Sigma) or IgM-HRP (Abcam) antibody diluted 1:10,000 in 4% goat serum. Following four washes, plates were incubated with UltraFast TMB ELISA substrate (ThermoFisher Scientific) followed by deactivation of the chromogenic reaction with sulfuric acid (2 m, Sigma). Samples and standards were run in duplicates. IgG and IgM concentration in serum was interpolated from the standard curve to which a 4PL nonlinear curve fit was applied using Prism (GraphPad) software.

#### T-lymphocytes and microglia/macrophages

T-lymphocyte infiltration and microglia/macrophages in the spinal cords of IgM-KO and WT mice were assessed by immunohistochemistry IHC at 2 and 10 weeks post-SCI in 11 sections per animal at 600-μm intervals.

For IHC staining, sections were first blocked in 5% goat serum + 1% BSA + 0.3% Triton X-100 in PBS. Next, sections were incubated with primary antibodies against the T-cell marker CD3 (goat anti-mouse CD3, 1:1000, Alexa Fluor 568, BioLegend) or the macrophage marker CD11b (rat anti-mouse CD11b, 1:300, Millipore CBL1313) overnight at 4°C. DAPI (1:250) was incubated with the anti-CD3 primary antibody or with the secondary antibody in sections stained for CD11b (goat anti-rat IgG, 1:500, Alexa Fluor 488, Life technologies), also overnight at 4°C.

Following imaging with a Nikon Eclipse E800 microscope at 10× magnifications, CD3+ or CD11b+ immunofluorescent areas or cell counts were estimated by ImageJ.

#### Myelin-engulfing macrophages

Myelin-engulfing macrophages were assessed in sections with the most intense IgG deposition (−1200, −600, and +600 μm from the injury epicenter). To this end, the selected sections were co-stained for fluoromyelin and CD11b. First, sections were stained for CD11b (primary + secondary antibody) as described above. Next, following washes, sections were incubated with fluoromyelin (1:100, Life Technologies, F34652) and DAPI (1:250) in 5% goat serum + 1% BSA + 0.3% Triton X-100 in PBS, overnight. Images were acquired with a Nikon eclipse Ti C2+ inverted confocal microscope under identical settings for each marker. The area of positive immunofluorescence for each marker was estimated with ImageJ and expressed as % of the total DAPI+ area of the spinal cord section. Here, we report averaged values that were obtained from three sections (representing distances −1200, −600, and +600 μm from the injury epicenter) per animal.

#### Experimental design and statistical analysis

Sample sizes for all experiments in this study were determined a priori based on previous studies with similar animal models and experimental design (Forgione et al., 2017; Ulndreaj et al., 2017). Considering the predefined sample size, samples that were assessed were selected randomly from the pool of all samples collected. All experimental assessments, data collection and analyses were performed by experimenters who were blinded to the study groups. All ImageJ analyses were performed in an automated fashion to minimize human bias.

Prism (GraphPad) and SPSS (IBM) were used for hypothesis-testing statistical analyses of all the data. Comparisons between two groups were conducted using Student’s *t* tests for parametric data, and Mann–Whitney tests for non-parametric data. Statistical comparisons for more than two groups were conducted with one-way ANOVA and Bonferroni *post hoc* tests for parametric data or with Kruskal–Wallis tests for non-parametric data, respectively. All tests were two-tailed and statistically significant differences were considered to be those with a *p* ≤ 0.05. CatWalk data were first analyzed by repeated measures to reveal changes between groups over time (Table 1). Parameters that were shown by repeated measures tests to differ significantly between groups were further analyzed by one-way ANOVA and Bonferroni *post hoc* tests to compare groups within each time point. Results are shown as individual data points, with each data point representing one animal. Bars indicate mean ± standard error of the mean (SEM) unless otherwise specified. Results from hypothesis testing statistical analyses are reported in the statistical table (Table 1).

**Table 1:** Hypothesis testing statistics table showing the statistical tests used in the study and respective results

Figure	Panel	Measure	Groups (sample size)	Test	Details	Statistic	*p* value
1	*B*	Effect of secretory IgM on the % area of IgG deposition at 2 weeks post-SCI	KO (4), WT (5) mice	Two-way ANOVA Sidak’s *post hoc* to compare between KO and WT mice for each distance point Factor 1 (genotype), factor 2 (distance from epileft)	Effect of genotype	*F* = 21.31	**<0.0001**
					Section −1200 μm	*t* = 3.528	**0.0078**
					Section −600 μm	*t* = 3.513	**0.0082**
					Section 600 μm	*t* = 2.957	**0.0445**
	*C*	Effect of secretory IgM on the % area of IgG deposition at 10 weeks post-SCI	KO (4), WT (4) mice	Two-way ANOVA Factor 1 (genotype), factor 2 (distance from epileft)	Effect of genotype	*F* = 1.965	0.1657
	*H*	Effects of secretory IgM on levels of serum IgG immunoglobulin	KO: BSL (9), 2 weeks (5), 10 weeks (7) mice	One-way ANOVA Holm–Sidak’s multiple comparisons test	One-way ANOVA between WT and KO	*F* = 6.694	**0.0002**
			WT: BSL (10), 2 weeks (6), 10 weeks (6) mice		BSL: (KO vs WT)	*t* = 0.760	0.6995
					2 weeks post-SCI: (KO vs WT)	*t* = 1.950	0.1662
					10 weeks post-SCI: (KO vs WT)	*t* = 0.330	0.7431
2	*B*	Effect of secretory IgM on the % area of CD3+ cell infiltration at 2 weeks post-SCI	KO (4), WT (5) mice	Two-way ANOVA Sidak’s *post hoc* to compare between KO and WT mice for each distance point Factor 1 (genotype), factor 2 (distance from epileft)	Effect of genotype	*F* = 15.93	**0.0001**
					Section −1200 μm	*t* = 2.689	**0.0088**
					Section −600 μm	*t* = 2.862	**0.0054**
					Section 0	*t* = 2.29	**0.0248**
					Section 600 μm	*t* = 2.776	**0.0069**
		Effect of secretory IgM on the % area of CD3+ cell infiltration at 10 weeks post-SCI	KO (4), WT (5) mice	Two-way ANOVA Factor 1 (genotype), factor 2 (distance from epileft)	Effect of genotype	*F* = 0.827	0.3659
	*C*	Effect of secretory IgM on CD3+ cell counts at 2 weeks post-SCI	KO (5), WT (5) mice	Two-way ANOVA Sidak’s *post hoc* to compare between KO and WT mice for each distance point Factor 1 (genotype), factor 2 (distance from epileft)	Effect of genotype	*F* = 9.394	**0.0029**
					Section −1200 μm	*t* = 2.000	**0.0486**
					Section −600 μm	*t* = 2.095	**0.039**
					Section 0	*t* = 2.275	**0.0253**
					Section 600 μm	*t* = 2.483	**0.0149**
		Effect of secretory IgM on CD3+ cell counts at 10 weeks post-SCI	KO (5), WT (5) mice	Two-way ANOVA Sidak’s *post hoc* to compare between KO and WT mice for each distance point Factor 1 (genotype), factor 2 (distance from epileft)	Effect of genotype	*F* = 7.335	**0.0081**
					Section −1800 μm	*t*= 2.351	**0.021**
3	*B*	Effect of secretory IgM on the % area of CD11b+ cells at 2 weeks post-SCI	KO (4), WT (5) mice	Two-way ANOVA Sidak’s *post hoc* to compare between KO and WT mice for each distance point Factor 1 (genotype), factor 2 (distance from epileft)	Effect of genotype	*F* = 20.69	**<0.0001**
					Section −1200 μm	*t* = 2.471	**0.0157**
					Section 0	*t* = 3.031	**0.0033**
					Section 600 μm	*t* = 2.800	**0.0065**
		Effect of secretory IgM on the % area of CD11b+ cell at 10 weeks post-SCI	KO (4), WT (5) mice	Two-way ANOVA Sidak’s *post hoc* to compare between KO and WT mice for each distance point Factor 1 (genotype), factor 2 (distance from epileft)	Effect of genotype No significant *post hoc* difference	*F* = 5.069	**0.0272**
	*C*	Effect of secretory IgM on CD11b+ cell counts at 2 weeks post-SCI	KO (5), WT (5) mice	Two-way ANOVA Sidak’s *post hoc* to compare between KO and WT mice for each distance point Factor 1 (genotype), factor 2 (distance from epileft)	Effect of genotype	*F* = 21.61	**<0.0001**
					Section −1200 μm	*t* = 2.096	**0.039**
					Section 600 μm	*t* = 2.566	**0.012**
		Effect of secretory IgM on CD11b+ cell counts at 10 weeks post-SCI	KO (5), WT (5) mice	Two-way ANOVA Sidak’s *post hoc* to compare between KO and WT mice for each distance point Factor 1 (genotype), factor 2 (distance from epileft)	Effect of genotype	*F* = 21.92	**<0.0001**
					Section −2400 μm	*t* = 2.573	**0.0117**
					Section −1200 μm	*t* = 2.421	**0.0175**
					Section −600 μm	*t* = 2.647	**0.0096**
					Section 1200 μm	*t* = 2.074	**0.041**
4	*B*	Effect of secretory IgM on % lesional tissue at 2 weeks post-SCI	KO (4), WT (5) mice	Two-way ANOVA Factor 1 (genotype), factor 2 (distance from epileft)	Effect of genotype	*F* = 2.681	0.1056
	*C*	Effect of secretory IgM on % white matter preservation at 2 weeks post-SCI	KO (4), WT (5) mice	Two-way ANOVA Factor 1 (genotype), factor 2 (distance from epileft)	Effect of genotype	*F* = 0.199	0.6566
	*D*	Effect of secretory IgM on % gray matter preservation at 2 weeks post-SCI	KO (4), WT (5) mice	Two-way ANOVA Fisher’s *post hoc* to compare between KO and WT mice for each distance point Factor 1 (genotype), factor 2 (distance from epileft)	Effect of genotype	*F* = 6.544	**0.0061**
				Section 0	*t* = 2.817	**0.0062**
	*E*	Effect of secretory IgM on % lesional tissue at 10 weeks post-SCI	KO (4), WT (5) mice	Two-way ANOVA Fisher’s *post hoc* to compare between KO and WT mice for each distance point Factor 1 (genotype), factor 2 (distance from epileft)	Effect of genotype	*F* = 22.53	**<0.0001**
					Section −2400 μm	*t* = 2.041	**0.0452**
					Section −1200 μm	*t* = 2.216	**0.0302**
					Section −600 μm	*t* = 3.328	**0.0014**
4	*F*	Effect of secretory IgM on % white matter preservation at 10 weeks post-SCI	KO (4), WT (5) mice	Two-way ANOVA Fisher’s *post hoc* to compare between KO and WT mice for each distance point Factor 1 (genotype), factor 2 (distance from epileft)	Effect of genotype	*F* = 33.38	**<0.0001**
					Section −2400 μm	*t* = 2.579	**0.0121**
					Section −1200 μm	*t* = 2.574	**0.0123**
					Section −60 μm	*t* = 2.918	**0.0048**
					Section 1800 μm	*t* = 2.496	**0.0151**
					Section 2400 μm	*t* = 2.331	**0.0228**
					Section 3000 μm	*t* = 2.028	**0.0466**
	*G*	Effect of secretory IgM on the % gray matter preservation at 10 weeks post-SCI	KO (4), WT (5) mice	Two-way ANOVA Factor 1 (genotype), factor 2 (distance from epileft)	Effect of genotype	*F* = 0.1437	0.7059
5	*C*	Effect of secretory IgM on step sequence regularity at 4, 8, and 10 weeks post-SCI		Three-way ANOVA One-way ANOVA for groups within each time point Sidak’s multiple comparisons *post hoc* test on one-way ANOVA Factor 1 (genotype), factor 2 (injury status), factor 3 (time post-SCI)	Effect of genotype × injury	*F* = 10.23	**0.0019**
			4 weeks: KO SCI (10), KO sham (10) WT SCI (7), WT sham (10) mice		4 weeks one-way ANOVA	*F* = 9.625	**0.0001**
					KO SCI vs KO sham	*t* = 3.985	**0.0011**
					KO SCI vs WT SCI	*t* = 0.427	0.9648
					WT SCI vs WT sham	*t* = 3.468	**0.0044**
			8 weeks: KO SCI (10), KO sham (8), WT SCI (7), WT sham (9) mice		8 weeks one-way ANOVA	*F* = 36.14	**<0.0001**
					KO SCI vs KO sham	*t* = 36.14	**<0.0001**
					KO SCI vs WT SCI	*t* = 3.675	**0.0028**
					WT SCI vs WT sham	*t* = 4.362	**0.0004**
			10 weeks: KO SCI (7), KO sham (8), WT SCI (6), WT sham (8) mice		10 weeks one-way ANOVA	*F* = 28.85	**<0.0001**
					KO SCI vs KO sham	*t* = 8.244	**<0.0001**
					KO SCI vs WT SCI	*t* = 4.415	**0.0005**
					WT SCI vs WT sham	*t* = 3.065	**0.0154**
	*D*	Effect of secretory IgM on hindlimb swing speed at 4, 8, and 10 weeks post-SCI		Three-way ANOVA One-way ANOVA for groups within each time point Sidak’s multiple comparisons *post hoc* test on one-way ANOVA Factor 1 (genotype), factor 2 (injury status), factor 3 (time post-SCI)	Effect of genotype × injury	*F* = 5.351	**0.0229**
			4 weeks: KO SCI (10), KO sham (10) WT SCI (7), WT sham (10) mice		4 weeks one-way ANOVA	F= 2.606	0.0682
					KO SCI vs KO sham	*t* = 2.535	**0.0477**
					KO SCI vs WT SCI	*t* = 1.015	0.682
					WT SCI vs WT sham	*t* = 0.979	0.7057
5	*D*	Effect of secretory IgM on hindlimb swing speed at 4, 8, and 10 weeks post-SCI	8 weeks: KO SCI (10), KO sham (8), WT SCI (7), WT sham (9) mice		8 weeks one-way ANOVA	*F* = 3.214	**0.0358**
					KO SCI vs KO sham	*t* = 2.423	0.0624
					KO SCI vs WT SCI	*t* = 2.372	0.0699
					WT SCI vs WT sham	*t* = 0.126	0.999
			10 weeks: KO SCI (9), KO sham (8), WT SCI (6), WT sham (8) mice		10 weeks one-way ANOVA	*F* = 5.733	**0.0127**
					KO SCI vs KO sham	*t* = 3.123	**0.0395**
					KO SCI vs WT SCI	*t* = 2.474	0.3076
			WT SCI vs WT sham		*t* = 1.04	0.668
	*E*	Effect of secretory IgM on forelimb swing speed at 4, 8, and 10 weeks post-SCI		Three-way ANOVA One-way ANOVA for groups within each time point Sidak’s multiple comparisons *post hoc* test on one-way ANOVA Factor 1 (genotype), factor 2 (injury status), factor 3 (time post-SCI)	Effect of genotype × injury	*F* = 0.998	0.3205
			4 weeks: KO SCI (10), KO sham (10), WT SCI (7), WT sham (10) mice		4 weeks one-way ANOVA	*F* = 5.912	**0.0024**
					KO SCI vs KO sham	*t* = 3.467	**0.0044**
					KO SCI vs WT SCI	*t* = 0.139	0.9987
					WT SCI vs WT sham	*t* = 2.337	0.075
			8 weeks: KO SCI (12), KO sham (8), WT SCI (7), WT sham (9) mice		8 weeks one-way ANOVA	*F* = 9.014	**0.0002**
					KO SCI vs KO sham	*t* = 4.364	**0.0004**
					KO SCI vs WT SCI	*t* = 0.107	0.9994
			WT SCI vs WT sham			*t* = 2.823	**0.0242**
			10 weeks: KO SCI (9), KO sham (8), WT SCI (6), WT sham (8) mice		10 weeks one-way ANOVA	*F* = 6.61	**0.0017**
					KO SCI vs KO sham	*t* = 3.418	**0.006**
					KO SCI vs WT SCI	*t* = 0.777	0.8278
			WT SCI vs WT sham			*t* = 2.595	**0.0446**
	*F*	Effect of secretory IgM on hindlimb print area at 4, 8, and 10 weeks post-SCI		Three-way ANOVA One-way ANOVA for groups within each time point Sidak’s multiple comparisons *post hoc* test on one-way ANOVA Factor 1 (genotype), factor 2 (injury status), factor 3 (time post-SCI)	Effect of genotype × injury	*F* = 1.416	0.2371
			4 weeks: KO SCI (10), KO sham (10), WT SCI (7), WT sham (10) mice		4 weeks one-way ANOVA	*F* = 0.641	0.5941
					KO SCI vs KO sham	*t* = 0.239	0.9934
					KO SCI vs WT SCI	*t* = 1.082	0.6375
					WT SCI vs WT sham	*t* = 1.095	0.6291
5	*F*	Effect of secretory IgM on hindlimb print area at 4, 8, and 10 weeks post-SCI	8 weeks: KO SCI (12), KO sham (8), WT SCI (7), WT sham (9) mice		8 weeks one-way ANOVA	*F* = 1.1	0.3635
					KO SCI vs KO sham	*t* = 0.340	0.9817
					KO SCI vs WT SCI	*t* = 1.393	0.4349
					WT SCI vs WT sham	*t* = 0.546	0.9306
			10 weeks: KO SCI (9), KO sham (8), WT SCI (6), WT sham (8) mice		10 weeks one-way ANOVA	*F* = 8.787	**0.0003**
					KO SCI vs KO sham	*t* = 2.31	0.0838
					KO SCI vs WT SCI	*t* = 4.399	**0.0005**
					WT SCI vs WT sham	*t* = 0.467	0.955
	*G*	Effect of secretory IgM on forelimb print area at 4, 8, and 10 weeks post-SCI		Three-way ANOVA One-way ANOVA for groups within each time point Sidak’s multiple comparisons *post hoc* test on one-way ANOVA Factor 1 (genotype), factor 2 (injury status), factor 3 (time post-SCI)	Effect of genotype × injury	*F* = 0.676	0.4132
			4 weeks: KO SCI (10), KO sham (10), WT SCI (7), WT sham (10) mice		4 weeks one-way ANOVA	*F* = 0.927	0.4387
					KO SCI vs KO sham	*t* = 0.808	0.8098
					KO SCI vs WT SCI	*t* = 0.968	0.7129
					WT SCI vs WT sham	*t* = 0.526	0.937
			8 weeks: KO SCI (12), KO sham (8), WT SCI (7), WT sham (9) mice		8 weeks one-way ANOVA	*F* = 3.558	**0.025**
					KO SCI vs KO sham	*t* = 0.661	0.8846
					KO SCI vs WT SCI	*t* = 1.111	0.6188
					WT SCI vs WT sham	*t* = 3.121	**0.0114**
			10 weeks: KO SCI (9), KO sham (8), WT SCI (6), WT sham (8) mice		10 weeks one-way ANOVA	*F* = 7.978	**0.0006**
					KO SCI vs KO sham	*t* = 2.636	**0.0406**
					KO SCI vs WT SCI	*t* = 1.231	0.5416
					WT SCI vs WT sham	*t* = 3.068	**0.0145**
	*H*	Effect of secretory IgM on average body speed at 4, 8, and 10 weeks post-SCI		Three-way ANOVA One-way ANOVA for groups within each time point Sidak’s multiple comparisons *post hoc* test on one-way ANOVA Factor 1 (genotype), factor 2 (injury status), factor 3 (time post-SCI)	Effect of genotype × injury	*F* = 0.558	0.457
			4 weeks: KO SCI (10), KO sham (10), WT SCI (7), WT sham (10) mice		4 weeks one-way ANOVA	*F* = 11.75	**<0.0001**
					KO SCI vs KO sham	*t* = 4.275	**0.0005**
					KO SCI vs WT SCI	*t* = 0.757	0.8374
					WT SCI vs WT sham	*t* = 4.099	**0.0008**
5	*H*	Effect of secretory IgM on average body speed at 4, 8, and 10 weeks post-SCI	8 weeks: KO SCI (12), KO sham (8), WT SCI (7), WT sham (9) mice		8 weeks one-way ANOVA	*F* = 13.56	**<0.0001**
					KO SCI vs KO sham	*t* = 5.539	**<0.0001**
					KO SCI vs WT SCI	*t* = 1.353	0.46
					WT SCI vs WT sham	*t* = 2.87	**0.0215**
			10 weeks: KO SCI (9), KO sham (8), WT SCI (6), WT sham (8) mice		10 weeks one-way ANOVA	*F* = 10.08	**0.0001**
					KO SCI vs KO sham	*t* = 3.972	**0.0014**
					KO SCI vs WT SCI	*t* = 0.678	0.8776
					WT SCI vs WT sham	*t* = 3.507	**0.0048**

*p* values <0.05 are shown in bold for emphasis.

**Figure 1.**
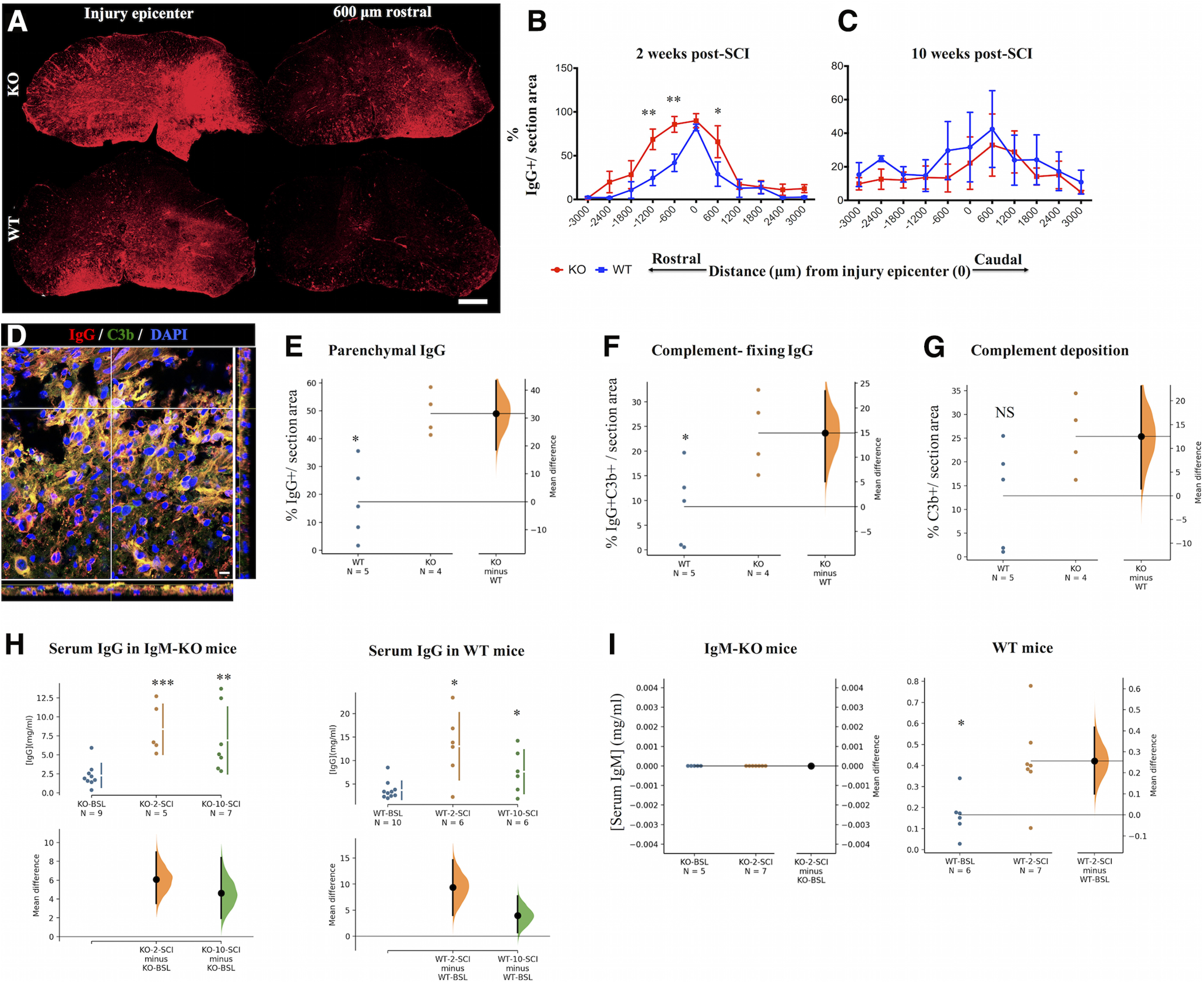
ΙgM KO mice have more parenchymal IgG antibodies with enhanced complement-fixing activity. ***A***, IgG deposition in spinal cord sections of IgM-KO and IgM-WT mice at two weeks post-SCI. Scale bar: 100 μm. ***B***, Comparison of the % detected IgG+ immunofluorescent area between groups at two weeks following SCI. See Table 1 for statistics details. ***C***, Comparison of the % detected IgG+ immunofluorescent area between groups at 10 weeks following SCI. See Table 1 for statistics details. ***D***, Representative section stained for IgG (red) and complement C3b protein (green). Yellow signal indicates co-localization of IgG and C3b. Scale bar: 10 μm. ***E–G***, Gardner–Altman estimation plots of % immunofluorescent area from deposited IgG, IgG+C3b, and C3b averaged between sections −1200, −600, and +600 μm from the injury epicenter, and normalized per section area (*N *=* *4–5 animals/group). See Table 2 for statistics details. ***H***, Gardner–Altman estimation plots of total serum IgG levels in IgM-KO and WT mice at BSL (pre-SCI) and at 2 and 10 weeks post-SCI. There is no difference in IgG levels between IgM-KO and WT mice within each time point (data not shown; see statistical Table 1 for details about WT vs KO comparisons). Serum IgG levels increase significantly after injury when compared with their BSL levels in IgM-KO and WT mice. See Table 2 for statistics details. ***I***, Gardner–Altman estimation plots of total serum IgM levels in IgM-KO and WT mice at BSL and at two weeks post-SCI, indicating a significant increase of IgM levels in WT mice, following SCI. See Table 2 for statistics details. NS = nonsignificant. Graphs ***B***, ***C***: mean ± SEM, *p <* *0.05, **p <* *0.01 (see Table 1 for detailed *p* values). Graphs ***E–I***: permutation *t* test, *p <* *0.05, **p <* *0.01, *** *p *<* *0.001 (see Table 2 for detailed *p* values).

Where possible, estimation statistical analyses reporting effect size, confidence intervals (CIs) and *p* values of permutation *t* tests, were performed according to (Ho et al., 2019) using the online tool (https://www.estimationstats.com). Briefly for these analyses, 5000 bootstrap samples were taken, and the CI was bias-corrected and accelerated. For each permutation *p* value, 5000 reshuffles of the control and test labels were performed. The *p* values reported are the likelihood of observing the corresponding effect size, if the null hypothesis of zero difference is true. In this study, statistical significance of the permutation *p* value was set to 0.05 and therefore, in the graphs of data where permutation *p* values were <0.05, the significant difference is indicated accordingly with asterisks (Figs. 1*E–I*, 3*E*, 5*A*,*C–H*, 6*B–E*). Table 2 summarizes the effect size (unpaired mean difference) of the difference between compared groups and the 95% CI with lower and upper bound, as well as the *p* values of permutation tests. Gardner–Altman estimation plots were generated using the online tool (https://www.estimationstats.com) where possible (Figs. 1*E–I*, 3*E*, 5*A*, 6*B–E*). Such plots depict the compared groups and the mean difference between the groups as a bootstrap sampling distribution. The mean difference is depicted as a dot; the 95% CI is indicated by the ends of the vertical error bar. Of note, CatWalk data were analyzed by both types of statistical approaches. Three-way ANOVA with *post hoc* test was performed to look at the effect of genotype, time point and injury status on the selected CatWalk parameters (Table 1), whereas estimation statistics were used to compare the WT-SCI and KO-SCI only, within each time point. CatWalk data were not graphed as Gardner–Altman plots for aesthetic reasons. Asterisks on the graphs representing CatWalk data refer to significant results from estimation statistics comparing WT-SCI and KO-SCI within each time point of the study (Table 2).

**Table 2:** Estimation statistics table showing effect size (unpaired means difference) with 95% CIs and permutation tests *p* values as follows: effect size [CI width with lower bound, upper bound]

Figure	Panel	Measure	Groups (sample size)	Effect size [CI width]	Permutation *p* value
1	*E*	Effect of secretory IgM on the % area of IgG deposition at 2 weeks in select sections (−1200, −600, 600 μm).	KO (4 mice, 3 sections each)	31.6 [95%CI 18.6, 43.4]	**0.0108**
			WT (5 mice, 3 sections each)		
	*F*	Effect of secretory IgM on the % area of IgG and C3b co-localization at 2 weeks in select sections (−1200, −600, 600 μm).	KO (4 mice, 3 sections each)	14.9 [95%CI 5.07, 23.4]	**0.03**
			WT (5 mice, 3 sections each)		
	*G*	Effect of secretory IgM on the % area of C3b deposition at 2 weeks in select sections (−1200, −600, 600 μm).	KO (4 mice, 3 sections each)	12.5 [95%CI 1.48, 23.1]	0.116
			WT (5 mice, 3 sections each)		
	*H*	Effects of secretory IgM on levels of serum IgG immunoglobulin	KO: BSL (9), 2 weeks (5), 10 weeks (7) mice	KO-2-SCI minus KO-BSL: 6.09 [95%CI 3.51, 8.99]	**0.0004**
				KO-10-SCI minus KO-BSL: 4.62 [95%CI 1.95, 8.39]	**0.004**
			WT: BSL (10), 2 weeks (6), 10 weeks (6) mice	WT-2-SCI minus WT-BSL: 9.36 [95%CI 4.02, 14.6]	**0.0016**
				WT-10-SCI minus WT-BSL: 3.96 [95%CI 0.703, 7.71]	**0.0228**
	*I*	Levels of serum IgM immunoglobulin in WT mice at BSL (pre-SCI) and 2 weeks post-SCI	WT: BSL (6), 2 weeks (7) mice	0.256 [95%CI 0.1, 0.416]	**0.0146**
3	*E*	Effect of secretory IgM on expansion of myelin engulfing macrophages in spinal cord sections with largest IgG deposition.	KO (4 mice, 3 sections each)	4.24 [95%CI 1.07, 7.19]	**0.0246**
			WT (5 mice, 3 sections each)
5	*A*	Effect of secretory IgM on BMS scores at 2 weeks post-SCI	KO (7), WT (7) mice	−0.286 [95%CI −1.86, 0.643]	0.575
		Effect of secretory IgM on grip strength at 2 weeks post-SCI	KO (7), WT (7) mice	3.74 [95%CI −6.4, 13.3]	0.485
5	*C*	Effect of secretory IgM on step sequence regularity at 4, 8, and 10 weeks post-SCI	4 weeks: KO SCI (10), KO sham (10), WT SCI (7), WT sham (10) mice	KO SCI vs WT SCI −3.95 [95%CI −23.1, 16.8]	0.739
			8 weeks: KO SCI (10), KO sham (8), WT SCI (7), WT sham (9) mice	KO SCI vs WT SCI −22.5 [95%CI −38.6, −8.21]	**0.0196**
			10 weeks: KO SCI (7), KO sham (8), WT SCI (6), WT sham (8) mice	KO SCI vs WT SCI 31.4 [95.0%CI −49.4, −12.2]	**0.0188**
	*D*	Effect of secretory IgM on hindlimb swing speed at 4, 8, and 10 weeks post-SCI	4 weeks: KO SCI (10), KO sham (10), WT SCI (7), WT sham (10) mice	KO SCI vs WT SCI −7.22 [95%CI −20.9, 4.97]	0.358
			8 weeks: KO SCI (12), KO sham (8), WT SCI (7), WT sham (9) mice	KO SCI vs WT SCI −16.1 [95%CI −34.6, −4.95]	0.0598
			10 weeks: KO SCI (9), KO sham (8), WT SCI (6), WT sham (8) mice	KO SCI vs WT SCI −18.2 [95%CI −32.9, −3.69]	**0.0412**
	E	Effect of secretory IgM on forelimb swing speed at 4, 8, and 10 weeks post-SCI	4 weeks: KO SCI (10), KO sham (10), WT SCI (7), WT sham (10) mice	KO SCI vs WT SCI 0.859 [95%CI −11.3, 12.7]	0.896
			8 weeks: KO SCI (12), KO sham (8), WT SCI (7), WT sham (9) mice	KO SCI vs WT SCI −0.507 [95%CI −8.66, 9.56]	0.925
			10 weeks: KO SCI (9), KO sham (8), WT SCI (6), WT sham (8) mice	KO SCI vs WT SCI −4.87 [95.0%CI −20.5, 7.37]	0.524
	*F*	Effect of secretory IgM on hindlimb print area at 4, 8, and 10 weeks post-SCI	4 weeks: KO SCI (10), KO sham (10), WT SCI (7), WT sham (10) mice	KO SCI vs WT SCI −0.0616 [95%CI −0.175, 0.08]	0.425
			8 weeks: KO SCI (12), KO sham (8), WT SCI (7), WT sham (9) mice	KO SCI vs WT SCI −0.037[95%CI −0.0826, 0.024]	0.289.
5	*F*	Effect of secretory IgM on hindlimb print area at 4, 8, and 10 weeks post-SCI	10 weeks: KO SCI (9), KO sham (8), WT SCI (6), WT sham (8) mice	KO SCI vs WT SCI −0.096 [95%CI −0.137, −0.0501]	**0.0006**
	*G*	Effect of secretory IgM on forelimb print area at 4, 8, and 10 weeks post-SCI	4 weeks: KO SCI (10), KO sham (10), WT SCI (7), WT sham (10) mice	KO SCI vs WT SCI −0.0352 [95%CI −0.115, 0.0438]	0.43
			8 weeks: KO SCI (12), KO sham (8), WT SCI (7), WT sham (9) mice	KO SCI vs WT SCI 0.0192 [95%CI −0.0109, 0.055]	0.352
			10 weeks: KO SCI (9), KO sham (8), WT SCI (6), WT sham (8) mice	KO SCI vs WT SCI −0.023 [95%CI −0.0664, 0.0159]	0.35
	*H*	Effect of secretory IgM on average body speed at 4, 8, and 10 weeks post-SCI	4 weeks: KO SCI (10), KO sham (10), WT SCI (7), WT sham (10) mice	KO SCI vs WT SCI 2.39 [95%CI −2.51, 8.17]	0.424
			8 weeks: KO SCI (12), KO sham (8), WT SCI (7), WT sham (9) mice	KO SCI vs WT SCI −3.52 [95%CI −7.53, 1.42]	0.171
			10 weeks: KO SCI (9), KO sham (8), WT SCI (6), WT sham (8) mice	KO SCI vs WT SCI −2.31 [95%CI −8.24, 3.55]	0.49
6	*B*	Effect of secretory IgM on bacterial load in bladder at 2 weeks post-SCI	KO (7), WT (7) mice	0.429 [95%CI 0.0, 1.29]	0
	*C*	Effect of secretory IgM on bacterial load in colon at 2 weeks post-SCI	KO (7), WT (6) mice Upon log-transformation: KO (5), WT (6) mice	1.29e+02 [95%CI −2.01e+02, 7e+02]	0.877
	*D*	Effect of secretory IgM on body weight at 2 weeks post-SCI	KO (7), WT (7) mice	−1.54 [95%CI −7.52, 3.85]	0.617
	*E*	Effect of secretory IgM on spleen weight at 2 weeks post-SCI	KO (7), WT (7) mice	0.005 [95%CI −0.0206, 0.0272]	0.686

*p* values for permutation tests <0.05 are shown in bold for emphasis.

## Results

### IgM-KO mice show increased deposition of complement-fixing IgG antibodies

Antibodies accumulate in the injured parenchyma during the subacute phase of cervical SCI in rats (Ulndreaj et al., 2017) and during the chronic phase of thoracic SCI in mice (Ankeny et al., 2009). Such antibodies have been shown to be autoreactive (Ankeny et al., 2006, 2009; Ulndreaj et al., 2017) and pathogenic by activating the classical complement system pathway (Ankeny et al., 2009). As IgM has been shown to regulate IgG autoimmunity under normal conditions (Boes et al., 2000), we hypothesized that IgM-KO mice would have increased IgG-antibody deposition in the lesioned spinal cord during the subacute phase of our cervical SCI model, compared with their WT counterparts.

In line with this hypothesis, we found enhanced IgG deposition in IgM-KO mice, as shown by increased %area of IgG+ immunofluorescence compared with WT mice (Fig. 1*A*,*B*; Table 1). Particularly at distances −1200, −600, and +600 μm from the injury epicenter, the deposited IgG was ∼125% higher in KO mice than in the WT group. However, the enhanced antibody accumulation did not appear to be sustained in the chronic phase of injury, as there was no significant difference in % IgG+ immunofluorescent area between injured IgM-KO and WT mice at 10 weeks post-SCI (Fig. 1*C*; Table 1).

To establish whether the IgG deposited in the spinal cord after injury could activate the complement system, we selected sections with the highest IgG accumulation (e.g., at distances −1200, −600, and +600 μm from the injury epicenter) and co-stained them for IgG and complement C3b protein (Fig. 1*D*). As expected, there was increased IgG deposition in IgM-KO mice in comparison to controls (Fig. 1*E*; Table 2). Moreover, the IgG-C3b co-stained area was significantly larger in IgM-KO mice than in WT controls with SCI, indicating enhanced complement-fixing activity of deposited IgG in the spinal cord of KO mice (Fig. 1*F*; Table 2). However, C3b deposition alone was not significantly different between IgM-KO mice and WT controls with SCI (Fig. 1*G*; Table 2) suggesting that lack of IgM does not affect complement deposition through all pathways, but rather complement deposition through the classical (antibody-mediated) pathway. Of note, one cannot exclude the possibility that lack of statistical difference (effect size 12.5, 95%CI: 1.48, 23.1, permutation *p *=* *0.116; Table 2) between the groups in Figure 1*G* could be because of increased variance in the WT samples.

To exclude Fc-mediated binding of the labeling anti-C3b antibody in the spinal cord, in our optimization assays we used a mix of Fc blocking peptides (Innovex, #NB30930) and compared binding of antibodies to untreated sections. However, we did not find a significant difference in binding of anti-C3b antibody between the samples treated with Fc block and the untreated samples, even in sections of high antibody binding such as the injury epicenter (data not shown). Thus, binding of the anti-C3b antibody in our spinal cord sections was not Fc-mediated.

Total levels of circulating IgG were similar between IgM-KO and WT mice at BSL (24 h before SCI) and at 2 and 10 weeks post-SCI (Fig. 1*H*; Table 1). This suggests that increased presence of parenchymal IgG deposition in IgM-KO mice at two weeks post-SCI is not because of higher circulating IgG levels in these mice. Moreover, IgG levels increased in both groups at 2 and 10 weeks post-SCI compared with their respective BSL levels (Fig. 1*H*; Table 2). Lastly, while there was no detectable IgM immunoglobulin in KO mice before and after the injury, serum levels of IgM increased at two weeks following SCI (Fig. 1*I*; Table 2).

### IgM-KO mice have more T-lymphocytes and microglia/macrophages with myelin-engulfing activity in the lesioned spinal cord

**Figure 2.**
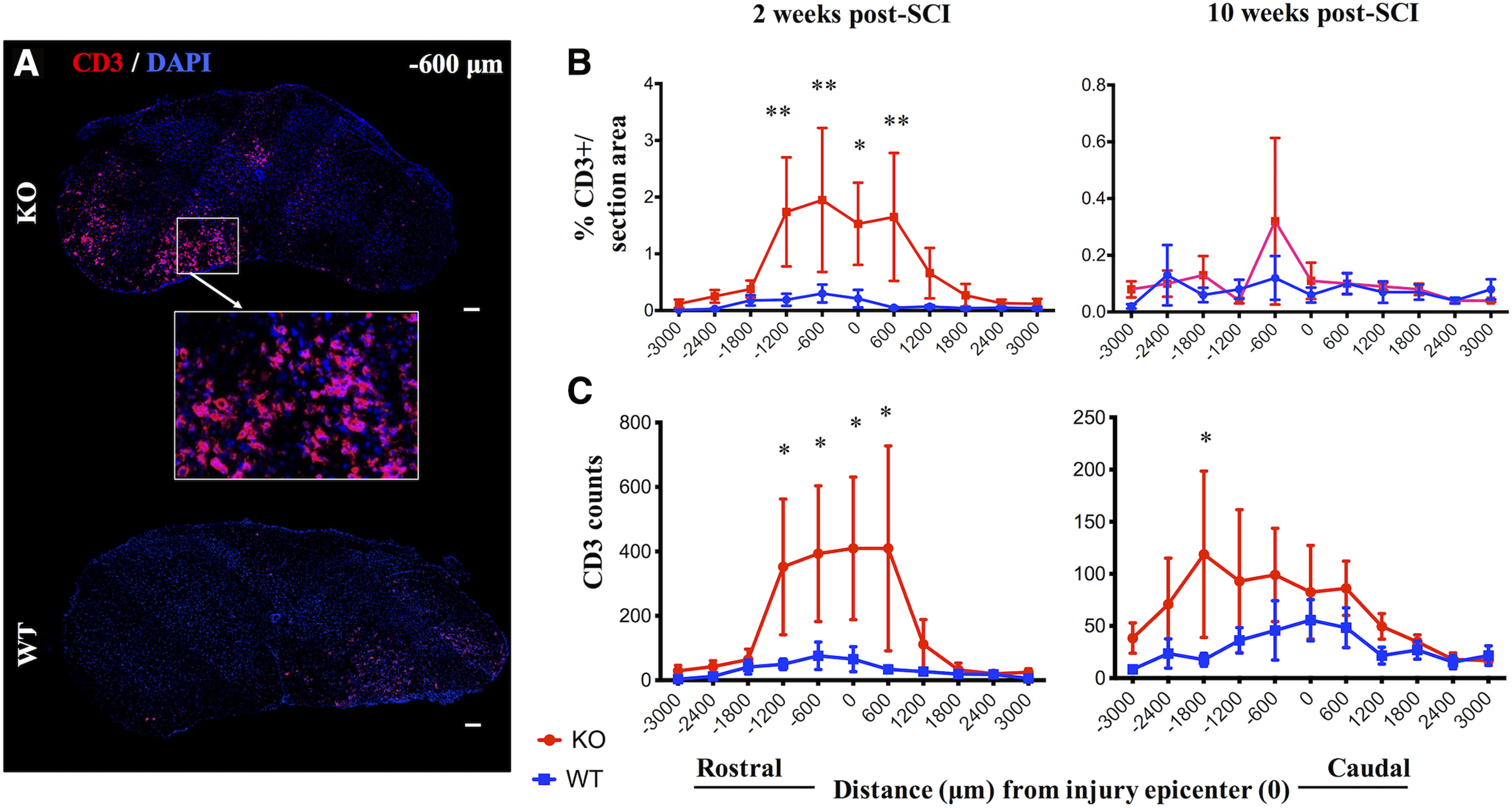
IgM-KO mice have increased T-lymphocyte infiltration compared with WT mice at two weeks post-SCI. ***A***, Representative sections stained for T-lymphocytes marker CD3 and DAPI in IgM-KO and WT mice at two weeks post-SCI. Quantification of % CD3+ immunostained area/total spinal cord section area (***B***) and CD3 cell counts (***C***) rostro-caudally from the injury epicenter, at 2 and 10 weeks post-injury in IgM-KO and WT mice. *N *=* *4–5 mice/group (***B***), 5 mice/group (***C***), mean ± SEM; *p <* *0.05, **p <* *0.01 (see Table 1 for detailed statistical results).

Inflammation mediated by microglia/macrophages and T-lymphocytes is a well-known regulator of pathophysiology in SCI (for review, see Donnelly and Popovich, 2008). Thus, we asked whether the absence of IgM in mice with SCI was linked to an enhanced presence of T-lymphocytes and microglia/macrophages. Indeed, IgM-KO mice showed larger CD3+ immunofluorescent areas and CD3+ cell counts, as compared with WT controls, at two weeks post-SCI (Fig. 2; Table 1). At 10 weeks post-SCI there was no difference in CD3+ immunofluorescence area between the IgM-KO and WT groups, but IgM-KO mice showed elevated CD3+ cell counts overall, and especially 1800 μm rostrally from the injury epicenter (Fig. 2*C*; Table 1).

**Figure 3.**
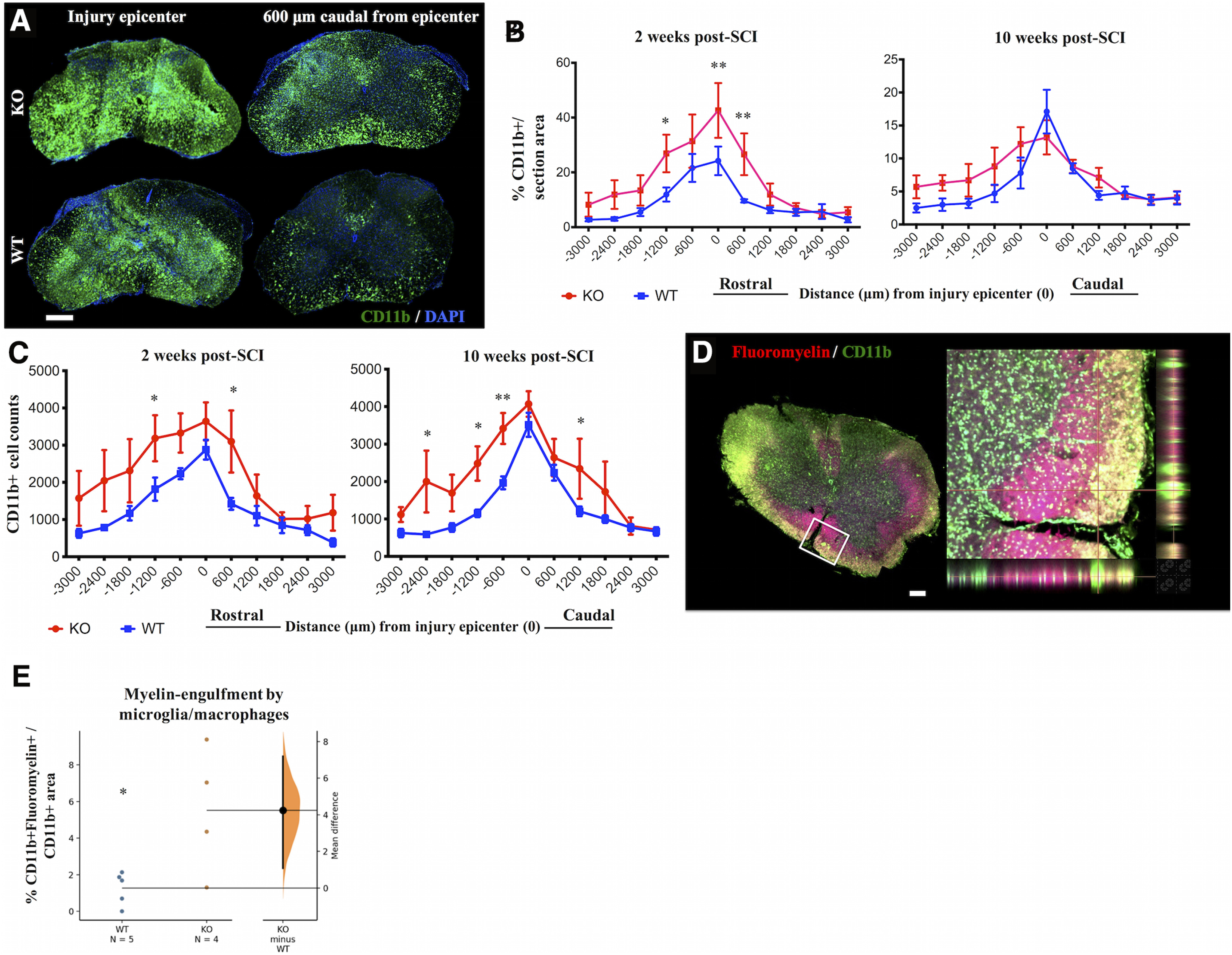
IgM-KO mice have more microglia/macrophages than WT controls. ***A***, Representative spinal cord sections stained for the microglia/macrophage markers CD11b and DAPI at two weeks post-SCI. Quantification of % CD11b+ immunostained areas/total area (***B***) and CD11b+ cell counts (***C***) rostro-caudally from the injury epicenter, at 2 and 10 weeks of injury. ***D***, Representative spinal cord section stained for myelin (fluoromyelin) and microglia/macrophages (CD11b) at two weeks post-SCI. Inset indicates area of co-localized CD11b and fluoromyelin signal. Myelin-engulfing macrophages appear yellow because of colocalization of green (CD11b+ signal) and red (fluoromyelin) signals in the single layer confocal image. ***E***, Gardner–Altman estimation plot showing quantification of % area of co-localized CD11b+ and fluoromyelin+ signal/total section area. Each dot indicates mean as averaged from 3 sections/animal, where sections were taken at distances −1200, −600, and +600 μm from the injury epicenter. Scale bar: 100 μm, *N *=* *4–5 animals/group (***B***, ***E***), 5 animals/group (***C***). Error bars indicate SEM; *p <* *0.05, **p <* *0.01. See Table 1 for detailed statistical results in ***B***, ***C*** and Table 2 for statistical results in ***E***.

In line with our hypothesis, we also found significantly larger CD11b+ %immunostained areas in IgM-KO mice than in WT mice at 2 weeks but not at 10 weeks (Fig. 3*A*,*B*; Table 1) of SCI. CD11b+ cell counts were higher in IgM-KO mice at both 2 and 10 weeks after injury (Fig. 3*C*; Table 1).

Next, we asked whether these microglia/macrophages had engulfed myelin, and whether there was a difference between IgM-KO mice and WT mice regarding the extent of myelin engulfment by microglia/macrophages. To this end, we semi-quantified the %area that was co-stained for CD11b and fluoromyelin (Fig. 3*D*) in sections with the highest IgG deposition (i.e., −1200, −600, and +600 μm from the injury epicenter) at two weeks post-SCI. Our data showed a significant increase in CD11b+fluoromyelin+ areas in the spinal cords of IgM-KO mice compared with WT controls at two weeks of SCI (Fig. 3*E*; Table 2), suggesting enhanced myelin engulfment by microglia/macrophages in the absence of IgM immunoglobulin.

### IgM-KO mice have larger lesion size in comparison to WT mice following SCI

**Figure 4.**
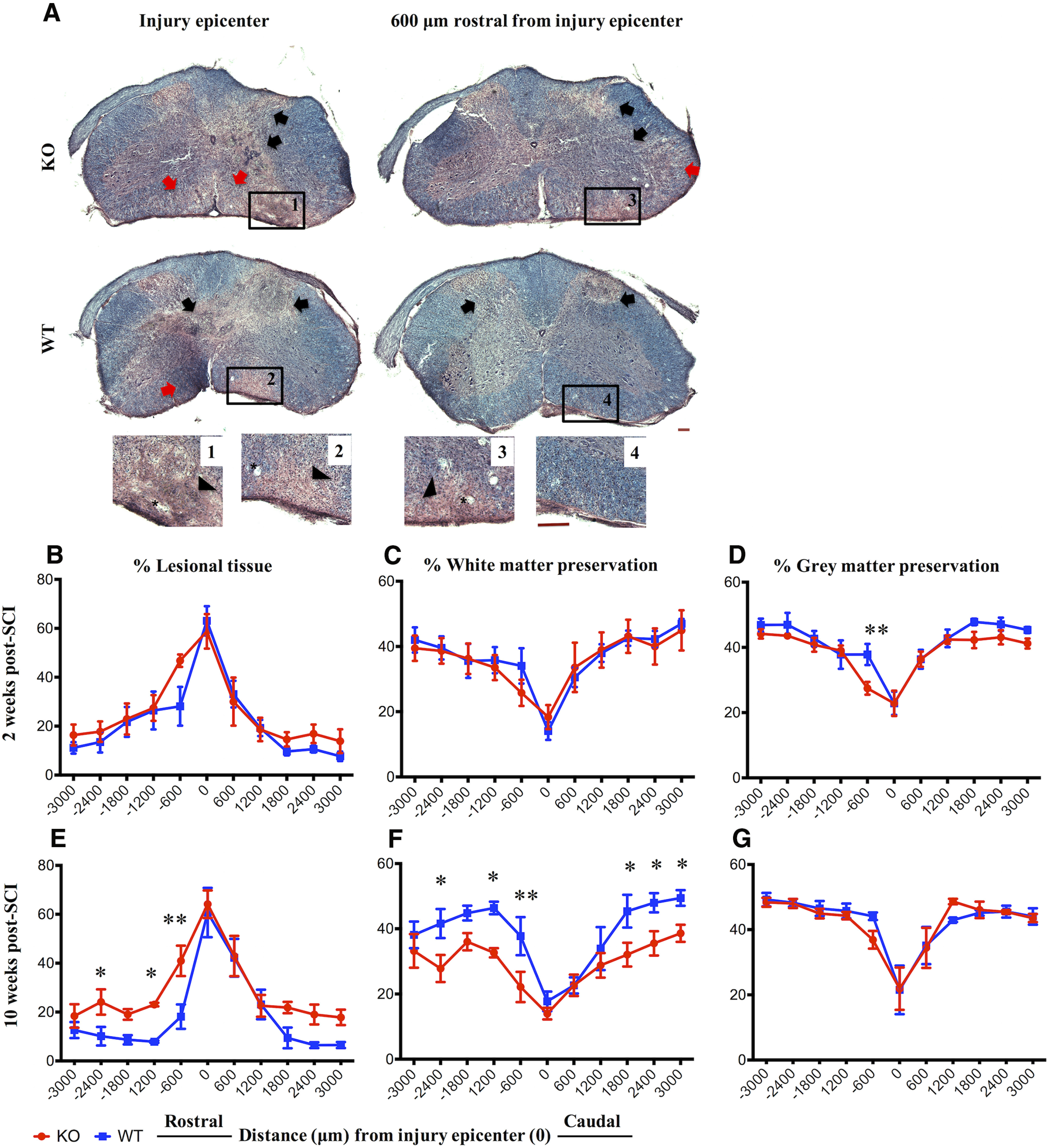
IgM KO mice have more lesional tissue at 10 weeks post-SCI. ***A***, Representative HE and LFB images of spinal cord cross-sections taken from an injured IgM-KO and WT mouse at the injury epicenter and 600 μm rostral from the injury epicenter at 10 weeks post-SCI. Insets 1–4 indicate most severe lesional tissue. Black arrows indicate gray matter lesions and red arrows indicate white matter lesions. Asterisks indicate vacuoles. Scale bar: 100 μm. ***B–G***, Quantification of lesional tissue and white/gray matter preservation by the Cavalieri method at 2 and 10 weeks post-SCI rostro-caudally from the injury epicenter. ***B–D***, Lesional tissue and white and gray matter preservation in IgM-KO and WT mice at two weeks of injury. ***E–G***, Lesional tissue and white and gray matter preservation at 10 weeks of injury *N *=* *4–5/group; mean ± SEM; *p <* *0.05, **p <* *0.01.

We quantified the volume of lesional tissue and white/gray matter sparing at 2 and 10 weeks post-SCI. Lesioned areas were present in both the gray and the white matter. In the gray matter, lesions had inflammation, apparent disruption of the normal cytoarchitecture along with fibrosis. In the white matter, lesions presented with inflammation, loss of LFB staining and vacuoles (Fig. 4*A*). There was no statistical difference between the groups in the volumes of lesional tissue (Fig. 4*B*; Table 1) or white matter preservation (Fig. 4*C*; Table 1) at two weeks of injury. However, the preserved gray matter was significantly lower in IgM KO mice, particularly at 600 μm rostral from the injury epicenter (Fig. 4*D*; Table 1).

Of note, IgM KO mice had significant loss of neural tissue at 10 weeks post-SCI. Compared with their WT counterparts, IgM-KO mice with SCI had more lesional tissue, especially rostral to the injury epicenter (Fig. 4*E*; Table 1). Concomitantly, IgM-KO mice had less white matter preservation rostrally and caudally from the injury epicenter (Fig. 4*F*; Table 1), while both groups had similar gray matter preservation (Fig. 4*G*; Table 1) at 10 weeks after injury.

### IgM deficiency results in impaired locomotor recovery during the chronic phase of SCI

**Figure 5.**
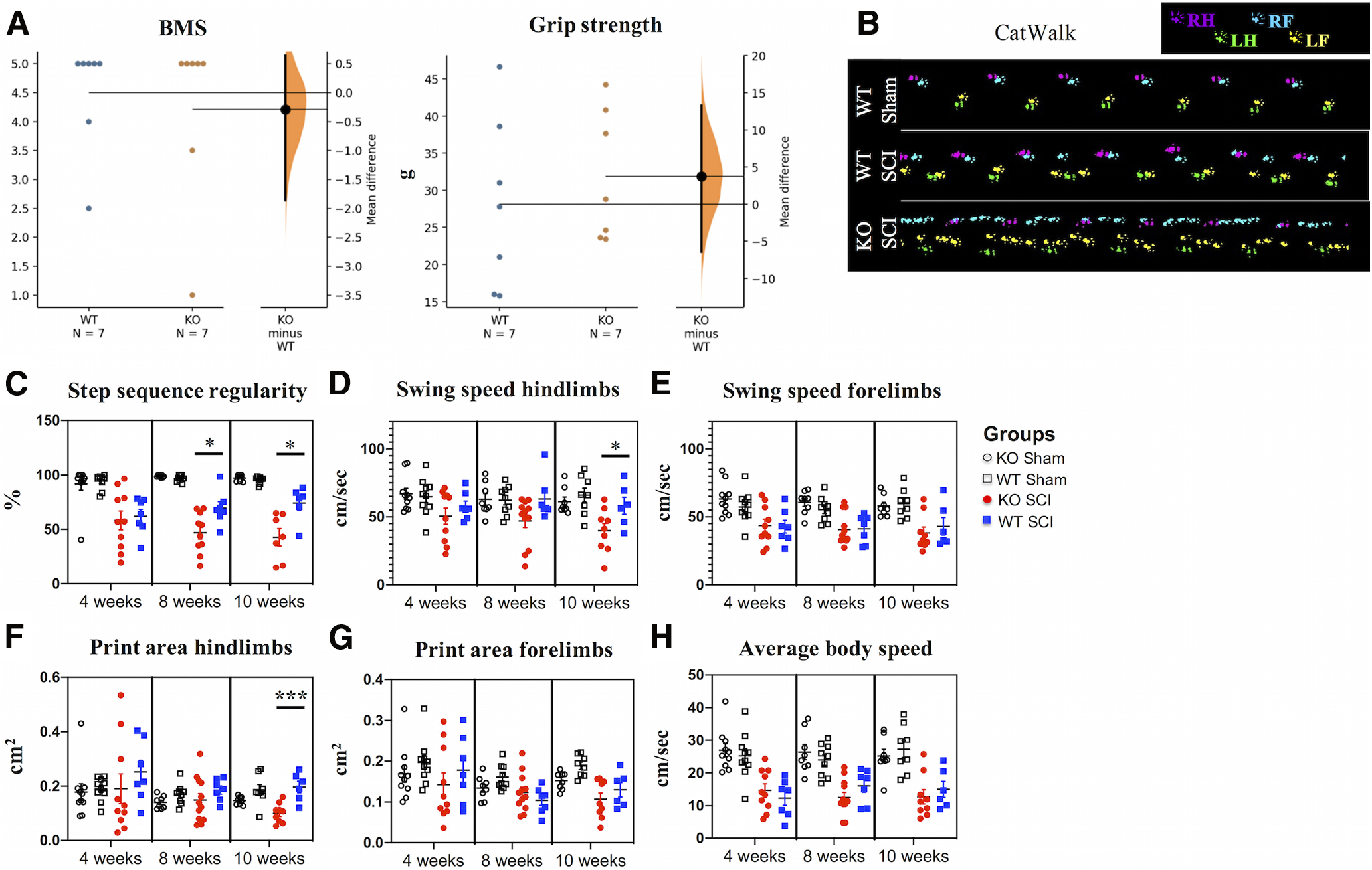
IgM deficiency results in impaired coordination during the chronic phase of SCI. ***A***, Gardner–Altman estimation plots of BMS and grip strength scores at two weeks after injury. See Table 2 for statistics details. ***B***, Representative images of locomotion in a sham WT animal as well as a WT and KO mouse with SCI at 10 weeks after injury, as recorded with CatWalk. ***C***, IgM-KO mice with SCI show deteriorated coordination compared with injured WT mice, as indicated by smaller step sequence regularity indices at 8 and 10 weeks after injury. See Table 2 for statistics details between WT and KO mice. ***D***, Hindlimb swing speed is slower in IgM-KO mice with SCI compared with their WT counterparts at 10 weeks of injury (Table 2). ***E***, There is no difference between WT and KO mice with SCI in forelimb swing speed (Table 2). ***F***, Hindlimb print area is significantly smaller in KO mice with SCI, compared with their WT counterparts (Table 2). ***G***, Forelimb print areas are similar between IgM-KO and WT mice with SCI (Table 2). ***H***, Average body speed is similar between IgM-KO and WT mice with SCI (Table 2). Group size: *N *=* *6–12 mice/group. Mean ± SEM, *p < 0.05, ***p < 0.001. Only significant differences between WT SCI and KO SCI groups are indicated with * on the graph, based on results shown in Table 2. For details on statistical analyses considering the effect of genotype, time after injury, and injury status on parameters shown in ***C–H***, see Table 1.

At two weeks post-SCI, IgM-KO mice and their WT counterparts showed similar locomotor and grip strength performance in BMS and grip strength tests, respectively (Fig. 5*A*; Table 2). However, at later time points, IgM-KO mice with SCI had impaired coordination and hindlimb function compared with their WT counterparts, based on their performance on the CatWalk test (Fig. 5*B*). In particular, injured KO mice had worse interlimb coordination compared with WT mice at 8 and 10 weeks post-SCI, as indicated by lower % step sequence regularity indices [Fig. 5*C*; Table 1 (for multiple comparisons across all time points and groups); Table 2 (for estimation statistics comparing WT-SCI to KO-SCI at each time point)]. By 10 weeks of injury, the difference between IgM-KO and WT mice with SCI was striking, with the regularity index ranging between 44–88% in WT mice, and 14–65% in KO mice.

Hindlimb swing speed was significantly impaired in KO mice compared with WT at 10 weeks of injury [Fig. 5*D*; Table 1 (for multiple comparisons across all time points and groups); Table 2 (for estimation statistics comparing WT-SCI to KO-SCI at each time point)]. Forelimb swing speed was significantly decreased in both genotypes after SCI when compared with their sham counterparts (Fig. 5*E*; Table 1). However, the absence of IgM did not appear to affect forelimb swing speed, as injured WT and IgM-KO mice showed similar recovery (Fig. 5*E*; Table 2).

IgM-KO mice had significantly smaller hindlimb print areas than their WT counterparts at 10 weeks after injury (Fig. 5*F*; Table 2). In both genotype groups, forelimb print area was reduced after injury when compared with their sham counterparts (Fig. 5*G*; Table 1). However, there was no difference in forelimb print areas between IgM-KO and WT mice with SCI (Fig. 5*G*; Table 2). Despite the above differences in coordination, hindlimb swing speed and hindlimb print area between the IgM-KO and WT mice with SCI, the average body speed was similar between IgM-KO and WT mice at all time points of the study [Fig. 5*H*; Table 1 (for multiple comparisons across all time points and groups); Table 2 (for estimation statistics comparing WT-SCI to KO-SCI at each time point)].

### IgM deficiency does not result in respiratory tract infections or urinary tract infections (UTIs)

**Figure 6.**
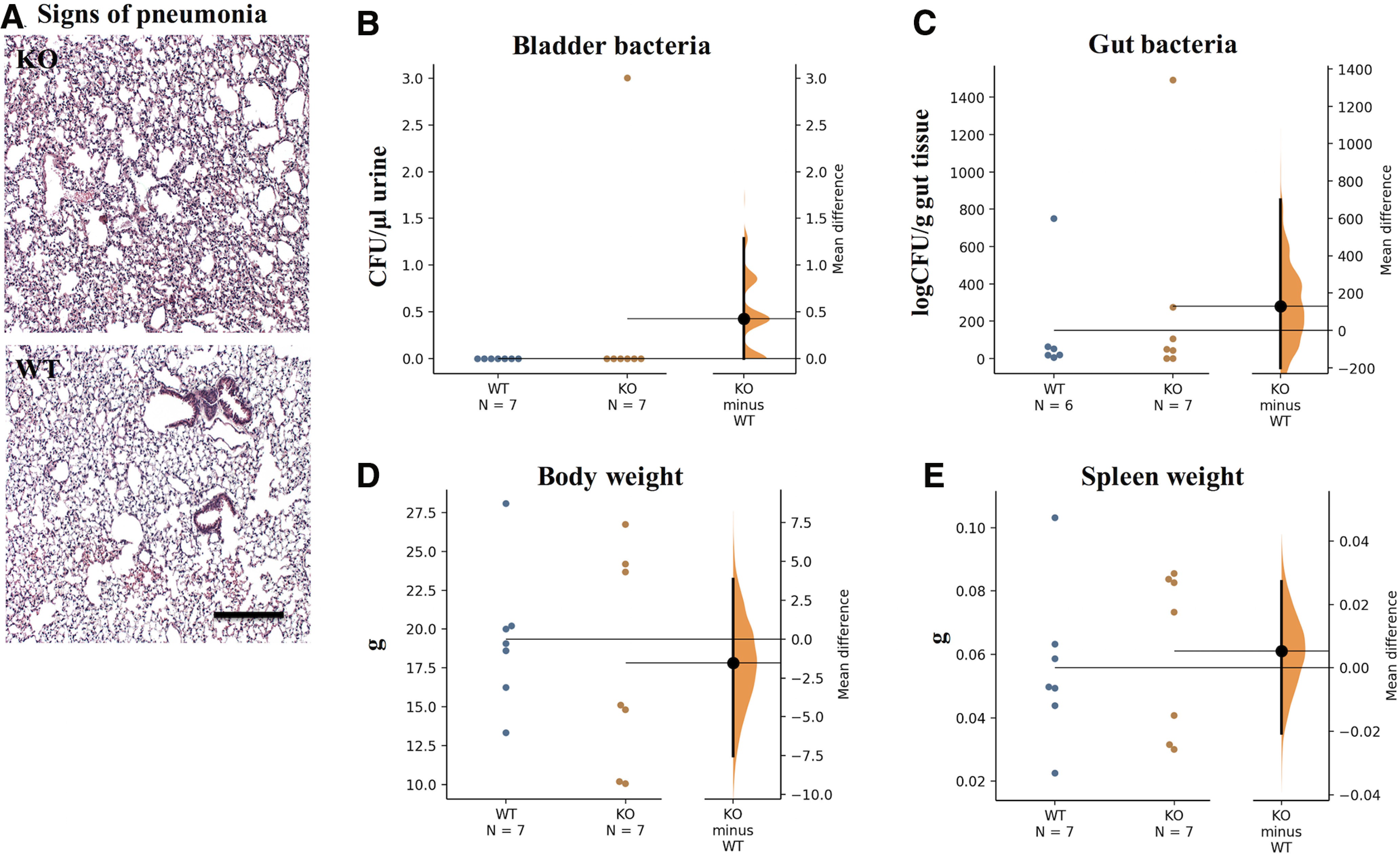
There is no evidence of bladder infection or pneumonia in IgM-KO mice or WT controls at two weeks of SCI. ***A***, HE-stained lungs at 4× magnification indicating absence of inflammation. ***B***, ***C***, Gardner–Altman estimation plots of bladder-bacterial and gut-bacterial load in IgM-KO and WT mice at two weeks of SCI. Gardner–Altman estimation plots of body weight (***D***) and spleen weight (***E***) in IgM-KO and WT mice at two weeks post-SCI. CFU = colony forming units; NS = nonsignificant. See Table 2 for detailed statistical results.

Infections have been shown to negatively affect neurologic recovery in SCI (Failli et al., 2012). Respiratory tract infections and UTIs are common complications in patients with SCI (Garcia-Arguello et al., 2017). As the peripheral immune response is particularly suppressed during the subacute phase of C7/T1 SCI in rats (Ulndreaj et al., 2017), we hypothesized that IgM-KO mice would have increased bacteria in their bladders and signs of pneumonia in their lungs at two weeks post-SCI. To test this hypothesis, we quantified the bacterial load in bladders of IgM-KO and WT mice with SCI and assessed for signs of inflammation in the lungs (e.g., abnormal presence of macrophages, increased numbers of macrophages and abnormal tissue structure) at two weeks after injury. There were no signs of lung inflammation in IgM-KO mice compared with WT mice (Fig. 6*A*). In addition, we found no significant increase in bacterial counts in the bladders of IgM-KO mice compared with WT mice with SCI (Fig. 6*B*; Table 2). Similar microbial counts were found in gut cultures (isolated from the colon), which were used as a positive control for detection and culturing of bacteria (Fig. 6*C*; Table 2). Other important metrics of recovery from SCI (Brommer et al., 2016) such as body weight (Fig. 6*D*; Table 2) and spleen weight (Fig. 6*E*; Table 2) were also similar between IgM-KO and WT mice at two weeks after injury.

### Discussion

This study shows that deficiency of IgM immunoglobulin results in impaired recovery following cervical SCI in mice. Overall, IgM-KO mice exhibited worse neurobehavioral recovery, coupled with increased lesion size, less white matter sparing, and enhanced deposition of complement-fixing IgG antibodies in the spinal cord as compared with their WT counterparts. These data provide evidence for the necessary role of IgM immunoglobulin in spontaneous recovery during cervical SCI and warrant further research into the therapeutic effect of IgM administration after SCI. A recent clinical trial showing significant benefits of IgM-enriched immunoglobulin preparation for patients with pneumonia and lower levels of endogenous IgM (Brunner et al., 2013; Cavazzuti et al., 2014; Wand et al., 2016; Welte et al., 2018; Kalvelage et al., 2019; Willuweit et al., 2019) further supports this notion.

Using a rat model of cervical SCI, we previously found early extravasation of IgM immunoglobulin in the lesioned spinal cord and a chronic expansion of splenic IgM plasma cells (Ulndreaj et al., 2017). Additionally, we have observed chronically increased levels of serum IgM immunoglobulin in rats after cervical SCI (Ulndreaj et al., 2020). Circulating IgM immunoglobulin comprises the majority of naturally occurring antibodies. By binding a broad range of self and non-self-targets, naturally occurring antibodies constitute a rapid innate response to altered self-antigens and invading pathogens, thereby maintaining homeostasis against autoimmunity, infectious and other diseases (Ehrenstein and Notley, 2010). Thus, we hypothesized that the enhanced IgM response we observed in our rat SCI model could be protective. To test this hypothesis we induced C6/C7 SCI in mice that lack secretory IgM immunoglobulin (Boes et al., 1998b) and compared their outcomes to WT littermate controls. These IgM-KO mice have undetectable serum IgM, although they express membrane IgM and all other secretory immunoglobulin classes (Boes et al., 1998b).

IgM-KO mice had significant deficits in coordination and other gait parameters in their hindlimbs compared with their WT counterparts during the chronic time points of our study (8–10 weeks post-SCI). When compared with shams, mice with SCI showed overall impairment in coordination, swing speed and print area in both fore- and hindlimbs, thus confirming the impairments previously observed in our C6/C7 SCI mouse model (Forgione et al., 2017). When comparing IgM-KO to WT mice with SCI, KO mice had greater impairments in coordination, lowered swing speed and smaller print area in the hindlimbs. Importantly, both groups had similar average body speeds, suggesting that the above differences did not result from the confounding effect of body speed (Batka et al., 2014).

In line with these chronic neurologic impairments, we found increased lesion size and less white matter sparing in IgM-KO mice compared with WT mice at our last chronic time point of the study (10 weeks after injury). Interestingly, both groups had similar neurobehavioral and histologic outcomes at two weeks of injury. This suggested that IgM deficiency may affect pathomechanisms that are initiated during or after the two-week time point, which marks the beginning of the subacute phase in rodent SCI.

One relevant mechanism explored here is the expansion of T cells and microglia/macrophages in the injured spinal cord. CD3 and CD11b cell expansion was assessed by quantifying the percent positive immunofluorescent area and by cell counts in spinal cord sections across a selected rostro-caudal axis from the injury epicenter. Of note, while microglia/macrophage responses are considered to peak by two weeks post-SCI in mice, this time point marks only the onset of T-cell infiltration in the lesioned spinal cord (Sroga et al., 2003). Also, in patients with SCI T-cell infiltrates are limited in the spinal cord and do not appear until weeks to months after trauma (Fleming et al., 2006). Given the decline in T-cell signal at 10 weeks, it is possible that here we have missed the peak of T-cell infiltration in the spinal cord. Despite this limitation regarding the optimal time point for the detection of CD3, our data show that compared with WT mice, IgM-KO mice had increased % CD3+ and % CD11b+ immunofluorescence area at two weeks post-SCI. At 10 weeks post-SCI, both groups had similar CD3 and CD11b + immunofluorescence areas. Interestingly, CD3 and CD11b cell counts were significantly higher in IgM-KO mice at both 2 and 10 weeks post-SCI. Our finding of higher CD3 and CD11b cell counts in IgM-KO mice with similar total immunofluorescence areas as WT mice at 10 weeks suggests that these cells in IgM-KO mice are on average smaller than in WT mice. This in turn can indirectly provide some information on the activation status of such cells or their subtype. For example, while both microglia and blood-derived macrophages are CD11b+, microglia tend to have a larger surface than blood-derived macrophages (Servet-Delprat et al., 2002). Similarly, IgM KO and WT mice may have different composition of CD3 cell subpopulations (CD4, CD8) with varying cell sizes depending on their activation state (Li and Kurlander, 2010). Taken together, these data show that CD3 and CD11b cells are expanded in the spinal cord of IgM-KO mice compared with their WT counterparts and further suggest that the composition of these cells is likely different between IgM-KO and WT mice.

Moreover, we found that IgM-KO mice had increased IgG antibody deposition in the lesioned spinal cord at two weeks of injury. We and others have previously observed extensive widespread IgG in the lesioned parenchyma in SCI models (Ankeny et al., 2009; Ulndreaj et al., 2017; Zhou et al., 2019). Such antibodies are localized with neurons (Ulndreaj et al., 2017), bind to astrocytes or astrocytic markers (Ulndreaj et al., 2017; Arevalo-Martin et al., 2018; Hergenroeder et al., 2018) or to vascular endothelial cells within the injured spinal cord parenchyma (Zhou et al., 2019). The deposited antibodies can be autoreactive [e.g., IgGs bind to spinal cord components through their F(ab’)_2_ portion] or not (e.g., IgGs bind to spinal cord cells through their Fc portion), and can persist throughout the disease course (Ankeny et al., 2009) or peak only during the subacute phase of SCI (Ulndreaj et al., 2017; Zhou et al., 2019). Regardless, there is strong evidence that IgG deposition in the spinal cord is detrimental to recovery after SCI (Ankeny et al., 2009; Zhou et al., 2019). Of note, circulating levels of IgG were similar between IgM-KO and WT mice at all time points of the study. This suggests that increased parenchymal IgG deposition at two weeks post-SCI in IgM-KO mice is likely not because of higher circulating IgG immunoglobulin. Rather, factors that affect IgG extravasation in the tissue [such as blood-spinal cord barrier (BSCB) disruption] and or/turnover are likely to contribute to enhanced IgG deposition in IgM-KO mice at two weeks post-SCI. The potential effect of IgM on BSCB integrity was outside the scope of this study. However, the accumulation of IgG, which appeared largely in areas mostly affected by the physical impact of clip compression (i.e., injury epicenter, anterior and lateral white matter), suggests that the degree of BSCB disruption may be different between IgM-KO and WT mice.

Here, we do not present direct evidence for the binding specificity of the IgG that was detected in the spinal cord. However, in our previous study we showed that circulating antibodies from rats with cervical SCI bind more strongly to cultured astrocytes (a cell type we found was IgG positive in injured spinal cords) than antibodies from uninjured rats. We also found that the spleen mounts a stronger antibody response against spinal cord antigens after injury. Furthermore, we performed competition assays to show that the signal we detect on the spinal cord is specific (Ulndreaj et al., 2017). These indirect lines of evidence suggest that some of the serum antibodies in mice with SCI are specific to spinal cord antigens. Others have also shown that SCI results in the induction of self-reacting antibodies (Ankeny et al., 2006, 2009). It should be noted that these induced antibodies comprise the minority of circulating antibodies (in the range of μg/ml) and we do not think that the signal we see in the spinal cord results exclusively from induced antibodies. Rather, given the large signal detected, we think it represents binding of naturally occurring antibodies that comprise the majority of the antibody pool in the body (in the mg/ml range), and typically bind to self-antigens with low affinity (Lutz, 2007). Such antibodies are elevated following SCI (Arevalo-Martin et al., 2018). Moreover, it has been shown that in the absence of circulating IgM immunoglobulins, there are more self-reacting IgG antibodies (Boes et al., 2000). Taken together, we think the deposition of IgGs in the injured spinal cord in the present study results from at least three non-mutually exclusive phenomena: (1) the BSCB disruption which allows for serum IgGs to enter the parenchyma non-specifically; (2) the binding of naturally occurring antibodies to spinal cord self-antigens with low affinity; (3) the generation of immune auto-IgGs following SCI and potential exacerbation of this response because of the absence of secretory IgM (Boes et al., 2000) specifically.

The histology and inflammation data taken together indicate that enhanced neuroinflammation (rather than worse lesion) during the subacute phase (two weeks post-SCI) is linked to chronic neurobehavioral impairments in IgM-KO mice. This observation is in line with multiple studies linking early neuroinflammation to deteriorated histologic and locomotor outcomes in the chronic phase (Bao et al., 2011; Nguyen et al., 2012; Brennan et al., 2015). It is unclear why IgM-KO and WT mice have similar lesion size at two weeks despite significant differences in neuroinflammation at the same time point. However, a plausible explanation could be that the enhanced inflammation we observed in IgM-KO mice has long-term functional effects that have not been established by two weeks after injury. These effects could be related to processes including regeneration and plasticity (Ballermann and Fouad, 2006; Courtine et al., 2008; Rossignol and Frigon, 2011), and a favorable balance of remyelination/demyelination (Hesp et al., 2015). Such mechanisms, although initiated early on during SCI, take longer to establish and to give rise to quantifiable differences in neurobehavioral outcomes.

One of the mechanisms by which deposited IgGs have been shown to negatively impact outcomes after SCI is through activation of the complement system (Ankeny et al., 2009). In turn, complement activation enhances immune cell infiltration/activation (such as microglia/macrophages and T-lymphocytes; Ankeny et al., 2009; Brennan et al., 2015) and promotes degradation of spared tissue by phagocytosis (Ankeny et al., 2009) or by direct complement-mediated attack (Qiao et al., 2010). Here, we examined whether increased IgG deposition in IgM-KO mice led to enhanced complement fixation in the lesioned spinal cord. In line with this hypothesis, we found enhanced C3b-IgG co-localization in the spinal cord of IgM-KO mice, as compared with their WT counterparts. Interestingly, C3b opsonizes antigens that are further taken up and engulfed by phagocytic cells (such as microglia and macrophages) through the interaction of C3b and complement receptor 3 (CR3), consisting of CD11b and CD18 (Ricklin et al., 2010). In an effort to understand the source of increased myelin loss in IgM-KO mice with SCI, and given the widespread presence of CD11b+ cells (typically microglia/macrophages) at two weeks of injury in KO mice, we further hypothesized that myelin was engulfed by CD11b+ microglia/macrophages in IgM-KO mice. Indeed, by quantifying the CD11b+/fluoromyelin+ area in sections with maximum IgG deposition (shown to have increased C3b deposition), we found that IgM-KO mice had significantly more myelin-engulfing microglia/macrophages than their WT counterparts. Taken together, this line of evidence indicates that the absence of secretory IgM immunoglobulin is associated with increased complement-fixing IgGs on the lesioned spinal cord, as well as the widespread presence of myelin-engulfing microglia/macrophages.

Research indicates that secretory IgM protects against detrimental IgG autoimmunity in multiple potentially overlapping pathways, which range from regulating the development of IgG autoantibodies to directly inhibiting their effector functions. For example, IgM antibodies enhance the phagocytic clearance of apoptotic cells and other cellular debris (Chen et al., 2009), which if left uncleared, could trigger pathogenic autoimmune responses (Henson, 2017). In addition, IgM regulates the development of pathogenic IgG autoantibody responses by controlling the generation of autoreactive B cells throughout their life span. Such mechanisms include central tolerance to self-antigens early during B cell development (Nguyen et al., 2015), and later on the reduction of autoreactive BCR signaling (Tsiantoulas et al., 2017) and deletion/deactivation of self-reactive B cells (Ouchida et al., 2012). Moreover, IgM immunoglobulin inhibits IgG autoantibody effector functions by binding directly to IgG’s F(ab’)_2_ component, thereby inhibiting IgG binding to self-antigens (Adib et al., 1990). We tested the latter possibility, given that in a previous study we found significant deposition of IgM antibodies in the spinal cord following C7 SCI in rats (Ulndreaj et al., 2017). However, contrary to our rat data, we did not find any significant IgM deposition at 2 or 10 weeks of injury in injured WT mice (data not shown), suggesting that IgM does not inhibit IgG binding in the injured spinal cord.

Of note, IgM’s protective effects on SCI recovery could be independent of IgG. For example, IgM was shown to induce proliferation of oligodendrocyte-precursor cells (OPCs) via Fcα/μR signaling during brain development (Tanabe and Yamashita, 2018). Occurring within the first two weeks post-SCI, and primarily rostrally from the injury epicenter (Li and Leung, 2015), oligodendrogenesis is an endogenous regenerative process in rodent SCI (Zai and Wrathall, 2005; Lytle and Wrathall, 2007; Tripathi and McTigue, 2007; Lytle et al., 2009; Li and Leung, 2015) and has been a therapeutic target for multiple research groups aiming to reverse myelin loss after injury (Karimi-Abdolrezaee et al., 2006; Plemel et al., 2014; Papastefanaki and Matsas, 2015). Thus, future studies could investigate whether secretory IgM promotes endogenous OPC proliferation in SCI.

Importantly, circulating IgM is the carrier and regulator of AIM (also known as CD5L/Spalpha/API6; Hiramoto et al., 2018), a protein with debris clearance and macrophage survival function. In the absence of secretory IgM, circulating levels of AIM are undetectable and insufficient debris clearance was shown to lead to worsened disease progression in a model of acute kidney injury (Arai and Miyazaki, 2018). It is thus possible that worse outcomes observed in IgM-KO mice with SCI in the present study are because of insufficient AIM-mediated debris clearance. This possibility should be explored in future studies as AIM was shown to be a top gene upregulated by microglia/macrophages after SCI (Zhu et al., 2017), yet its role in SCI remains unknown.

In addition to the above positive effects, IgM protects against microbial infections. Here, we looked at a possible link between IgM deficiency and microbial infections after injury, as infections are linked with poor neurologic recovery in SCI (Failli et al., 2012; Brommer et al., 2016) and IgM-KO mice have been shown to be susceptible to microbial infections in past studies (Baumgarth et al., 2005). As UTIs and pneumonia are the most frequent infection types in patients with SCI (Garcia-Arguello et al., 2017), we looked at microbial loads in the bladder and signs of inflammation in the lungs at two weeks after injury. However, we found no signs of bladder infection or lung inflammation. Thus, our data suggest that the impaired recovery after SCI in the absence of secretory IgM immunoglobulin is not because of infections. However, it is important to note that our experiments were conducted in mice that were receiving antibiotics in their water and were infection free.

Although our data suggest that total IgM immunoglobulin may have a protective role in SCI, it is important to note that not all IgM is beneficial for SCI. For example, some natural IgM antibodies were shown to have significant detrimental effects on SCI outcomes by activating the immune system and further enhancing secondary injury (Narang et al., 2017). This duality of the IgM response during SCI reflects the overall complex role of the immune response in SCI pathophysiology, which has made the journey to discovering effective SCI therapies challenging. Delineating the role of critical pathophysiological factors in SCI recovery, such as important components of the immune system, will take us closer to developing effective treatments for this debilitating chronic condition, which given its complex nature, will ultimately necessitate the application of an array of treatment modalities (Ulndreaj et al., 2016).

In conclusion, the present study shows that IgM immunoglobulin is important for recovery following cervical SCI, as IgM deficiency resulted in impaired neurobehavioral and histologic outcomes, as well as increased IgG deposition, complement activation, T- cell infiltration and myelin-engulfing macrophages. We anticipate that future research will investigate whether circulating IgM levels are useful biomarkers of recovery in patients with SCI, and ultimately, whether modulating IgM immunoglobulin levels can improve recovery.

## Synthesis

Reviewing Editor: Aniko Korosi, University of Amsterdam Decisions are customarily a result of the Reviewing Editor and the peer reviewers coming together and discussing their recommendations until a consensus is reached. When revisions are invited, a fact-based synthesis statement explaining their decision and outlining what is needed to prepare a revision will be listed below. The following reviewer(s) agreed to reveal their identity: Martin Schwab. Note: If this manuscript was transferred from JNeurosci and a decision was made to accept the manuscript without peer review, a brief statement to this effect will instead be what is listed below.

The manuscript has been revised with additional data, parts of new figures and explanations and a more in-depth discussion. Most of the questions of this reviewer have been answered. The manuscript has been improved significantly.

A few minor points should be revised nevertheless:

ad point of criticism 1): If a quantification method yields a result that is clearly different from the visual impression obtained from the original data material, a closer look is strongly recommended. The area measurement used here in Fig. 1-3 does not take into account the intensity of staining. Accordingly, the result shown e.g. in Fig. 1B, which suggests that the “parenchymal IgG deposition” (legend to Fig. 1) is identical at the epicenter of the lesion in WT and KO mice, differs greatly from the pictures shown in the representative immunofluorescent sections of Fig. 1A. Area measurements are a fast and cheap method, but they do not reflect the IgG content. If the authors want to stick with this method, the legend and the results text have to be revised in order to not give the impression that contents are shown rather than areas of infiltrated spinal cord tissue.

ad point 6): The IgM serum data shown in the rebuttal letter figure are interesting and relevant. They should be shown to the readers, not only to the referee. They would fit nicely into Fig. 1 (as panel 1I).
